# Genetic and non-genetic factors affecting semen production and quality characteristics of Gir cattle breed under semi-arid climate

**DOI:** 10.14202/vetworld.2020.1714-1718

**Published:** 2020-08-27

**Authors:** K. G. Bhave, K. Thilak Pon Jawahar, P. Kumarasamy, T. Sivakumar, C. Joseph, T. Shirsath, P. Deshmukh, R. Venkataramanan

**Affiliations:** 1 Department of Animal Genetics and Breeding, Madras Veterinary College, Tamil Nadu Veterinary and Animal Sciences University, Chennai, Tamil Nadu, India; 2 Controller of Examinations, Madhavaram Milk Colony, Tamil Nadu Veterinary and Animal Sciences University, Chennai, Tamil Nadu, India; 3 Dean, Veterinary College and Research Institute, Orathanadu, Thanjavur, Tamil Nadu, India; 4 Director of Research, Madhavaram Milk Colony, Tamil Nadu Veterinary and Animal Sciences University, Chennai, Tamil Nadu, India; 5 Frozen semen laboratory, BAIF, Development Research Foundation, Dr. Manibhai Desai Nagar, Uruli Kanchan, Pune, Maharashtra, India; 6 Livestock Farm Complex, Madhavaram Milk Colony, Tamil Nadu Veterinary and Animal Sciences University, Chennai, Tamil Nadu, India

**Keywords:** Gir, longitudinal analysis, repeatability, bull effect, and semen production

## Abstract

**Aim:**

This study aims to evaluate genetic and non-genetic factors influencing semen production potential of Gir bulls.

**Materials and Methods:**

Data on semen quantity (n=6911) and quality (n=466) available from January 2011 to December 2018 at BAIF’s frozen semen station, Jind, Haryana, India, were utilized for the study. Factors, namely, season of collection, age at collection, and bull (random effect) were studied for their effect on quantitative and qualitative semen traits. Least square means for the traits were obtained using a general linear model. The effect of age within bull for repeatable traits was analyzed using a longitudinal model with age as the control variable. Multivariate analysis using mixed repeatability model equation was utilized to estimate bull effect correlation (genetic + permanent environmental correlation), phenotypic correlations, and repeatability.

**Results:**

The overall least square means of ejaculate volume, sperm concentration, total sperms, initial and post-thaw motility, hypo-osmotic swelling test, and acrosome integrity of frozen semen were 6.62±0.03 ml, 1.22±0.01 10^9^/ml, 8.09±0.05 10^9^/ejaculate, 75.78±0.001%, 55.92±0.0001%, 55.13±0.005%, and 71.08±0.001%, respectively. The season of the collection showed a significant effect on volume, concentration, total sperm, and initial motility. The performance of bulls was superior in summer season, followed by winter and monsoon. Increase in semen attributes during summer season was due to the effect of lower temperature on sensitive stages of spermatogenesis. Age at collection had a significant effect on all semen traits. Volume and total sperm count showed increasing trend while concentration showed a decreasing trend with an increase in age. Motility and quality traits did not show any particular pattern. Individual bulls showed differences in all the semen performance traits with age. The repeatability of the traits ranged from 0.04 (HOST) to 0.58 (acrosome integrity). Bull effect correlation ranged from −0.73 (initial motility and acrosome integrity) to 0.93 (HOST and acrosome integrity).

**Conclusion:**

Individual bulls showed variation in traits measured over age. The result of the study could be utilized in suggesting suitable management plans to achieve the desired profit by improving semen quality in Gir bulls.

## Introduction

Artificial insemination (AI) technique is considered a boon to the livestock industry. AI is a matter of great significance due to large-scale use of very few genetically superior sires to cover a large female population. As per the breeding policy of Government of India, selective breeding and upgrading of non-descript bovine population using frozen semen from indigenous cattle is recommended. Gir breed is a recognized milk purpose breed of India which has been extensively used for selective breeding and upgrading of non-descript cattle population. Gir cattle breed is also known for their heat tolerance resistance to disease and ability to survive on low-grade feeding resource.

As per the 20^th^ livestock census, the population of indigenous cattle has declined by around 6% [1] and there is an urgent need to increase the number of good quality semen doses from superior bulls. Quantity and quality of semen are sensitive to environmental factors. Information regarding effect of season and age at collection on semen characters of different indigenous breeds such as Nellore [2], Sahiwal [3], Red Sindhi [4], and Amritmahal [5] are available in literature. However, information on semen characteristics of Gir bulls is scarce. These studies available are limited to basic descriptive statistics and based on a small sample size. Moreover, there is a paucity of literature on genetic parameters of semen traits in Gir bulls.

The present study was undertaken to determine the effect of age and season on semen traits and to estimate their genetic parameters in Gir cattle breed.

## Materials and Methods

### Ethical approval

Ethical approval was not necessary for this study as no experimental animals were used.

### Data

Data on semen quantity (n=6911) and quality (n=466) of 38 Gir bulls, available from January 2011 to December 2018 at BAIF’s frozen semen station, Jind, Haryana, India, were utilized for the study.

### Farm location and climate

The BAIF frozen semen station is located in Haryana state, India, on the outskirts of Jind city (29.3159° N, 76.4896° E) at an altitude of 227 m above sea level. The region experienced three different seasons, namely, summer (March-May), monsoon (June-October), and winter (November to February). The mean minimum temperature ranged from 6.4°C in January to 17.3°C in October. The mean maximum temperature varies from 29.1°C in November to 40.9°C in May. The mean humidity varied from 23% in April to 78% in August. The region received an annual rainfall of 536 mm on average, mostly during the months of July-September. The average monthly rainfall ranged from 1 (April) to 178 (August) mm.

### Traits studied and influencing factors

The semen traits included in the study were ejaculate volume (ml), sperm concentration (10^9^/ml), initial and post-thaw motility (% of motile sperms after dilution and before/after thawing), total sperms (10^9^/ejaculate, as the product of volume and sperm concentration), hypo-osmotic swelling test (HOST), and acrosome integrity test frozen semen (%). The observations beyond mean±4 standard deviations were considered as outliers and removed from the study. The various factors influencing semen characteristics considered in the study were season (winter, summer, and monsoon) and age (classified as 12 months interval class, from 49 to 156 months) at collection.

### Semen processing for quality control

All the bulls were washed and cleaned early morning before semen collection. Handling and semen collection of a particular bull were performed by the same person responsible for that group of bulls. Dummy bulls were used for sexual stimulation and every bull was allowed 2-3 false mounts before actual semen collection. The time between false mounts varied among the bulls. One or two semen ejaculates were collected using an individual artificial vagina according to a standard procedure[6]. The collected semen in a glass tube was kept in a water bath for 37°C after recording the ejaculate volume. The sperm concentration was estimated by a digital photometer (IMV Technologies). The initial motility (percent progressive motile sperms) was assessed subjectively in diluted semen. The ejaculates which did not fulfill minimum criteria set by standards were removed from production, although the corresponding data were considered for the analysis. After the initial assessment, 0.25 ml semen doses were prepared with 20 × 10^6^ sperms per dose, sealed, and printed using IS4 instrument of IMV Technologies (L’Aigle, France). Semen straws were cooled to 4°C in 3 h and frozen step by step to reach −140°C in 7-8 min in a programmable freezer (IMV Technologies) followed by submersion and storage in liquid nitrogen at −196°C. Post-thaw motility assessment was then carried out 24 h post-freezing. Standardized initial and post-thaw motility assessments were separately carried out by different technicians. The assessment of mass activity, initial, and post-thaw motility was carried out using a phase contrast microscope (Nikon ECLIPSE E400, Tokyo, Japan). After cryopreservation of semen, random samples from a batch of collection from each bull were subjected to quality control testing. Quality control tests were carried out once in 3 months, allowing every bull to be tested 4 times a year. HOST was carried out using a quantity of 0.1 ml of frozen-thawed semen mixed with 1 ml of the hypo-osmotic solution and incubated at 37°C for at least 30 min. The stained smear later was observed using a phase contrast microscope to assess the “tail curling” appearance of the sperm. Intactness of acrosome was tested using acrosome integrity test with frozen semen which was carried out using Giemsa staining. The cutoffs for the mentioned tests were >40 and >65%, respectively.

### Statistical analysis

Before the analysis, percent data were transformed using the arcsine transformation. The semen production records were subjected to statistical analysis using least square analysis using “*lm*” function in R statistical software [7]. Along with “*lm*” function, “*car*,” “emmeans,” and “*agricolae*” packages were used for ANOVA (Type III sum of square) and Duncan’s multiple range test. The model used for the analysis can be represented as follows:

Y_ijk_ = μ + S_i_ + A_j_ + e_ijk_

Where,

Y_ijk_ = Semen trait

μ = Overall mean

S_i_ = Effect of the i^th^ season of collection (i = 1-3)

A_j_ = Effect of the j^th^ age at collection of a bull (j = 1-9)

e_ijk_ = Random error associated with the Y^ijk^ which is assumed to be normally and independently distributed with mean zero and constant variance

The longitudinal data analysis was carried out using “*lmerTest*” package in R. A linear mixed model with age and season of collection as fixed effects and age within bull as random effect was used. The significance of within bull variation was analyzed using a likelihood-ratio test by comparing the likelihoods of models with and without the within bull random effect. To study the bull effect correlation (genetic + permanent environmental correlation) on the semen parameters, a repeatability animal model with bull as a random effect was used in WOMBAT software [8].

## Results and Discussion

The results of least squares analyses are presented in Table-1. The mean ejaculate volume (6.62 ml) of Gir bulls observed in the present study was similar to earlier reports of Rana and Dhami [9] (7.03 ml), Das *et al*. [10] (6.2-6.4 ml), and Pathak *et al*. [11] (6.69 ml). The studies conducted by Shelke and Dhami [12], Sannat *et al*. [13], Sonar *et al*. [14], and Chikhaliya *et al*. [15] who reported comparatively lower ejaculate volumes (ranging from 4.5 to 5.80) in Gir.

**Table-1:** Least square means ± SE and number of observations for the effect of age and season of collection on semen traits of Gir bulls.

Levels of factors	VOL	CONC	TS	IM	PTM	HOST	AIFS
Overall means	6.62±0.03	1.22±0.01	8.09±0.05	75.78±0.001	55.92±0.0001	55.13±0.005	71.08±0.001
Season of collection	**	**	**	**	NS	NS	NS
Winter	6.83±0.15^b^	1.19±0.04^b^	8.12±0.29^b^	77.23±0.005^a^	55.92±0.001	55.57±0.01	74.55±0.009
Summer	6.98±0.16^a^	1.28±0.03^a^	8.97±0.30^a^	76.19±0.005^b^	55.74±0.001	55.22±0.01	73.93±0.01
Monsoon	6.72±0.15^b^	1.17±0.04^b^	7.78±0.29^b^	76.05±0.005^b^	55.92±0.001	54.70±0.009	73.16±0.001
Age at collection (months)	**	**	**	**	**	**	**
>48 ≤ 60	5.34±0.10^e^	1.33±0.02^a^	7.46±0.18^d,e^	77.37±0.001^a,b,c^	56.38±0.001^a^	52.79±0.01^b^	69.54±0.01^b^
>60 ≤ 72	5.64±0.06^e^	1.24±0.01^b,c,d^	7.16±0.12^e^	73.78±0.001^d^	55.27±0.001^c^	53.83±0.01^a,b^	70.50±0.01^b^
>72 ≤ 84	6.44±0.05^d^	1.30±0.01^b,c^	8.44±0.11^c^	75.45±0.001^c^	55.85±0.001^a,b^	56.61±0.01^a,b^	70.34±0.01^b^
>84 ≤ 96	6.79±0.07^c^	1.20±0.01^d,e^	8.26±0.14^c^	77.37±0.001^b^	56.2±0.001^a^	57.99±0.02^a,b^	72.08±0.02^b^
>96 ≤ 108	7.24±0.08^b^	1.11±0.01^e^	7.93±0.15^c,d^	78.39±0.001^a^	56.13±0.001^a,b^	58.34±0.01^a,b^	70.81±0.02^b^
>108 ≤ 120	7.79±0.11^a^	1.21±0.02^c,d^	9.18±0.20^b^	77.95±0.002^c^	55.43±0.001^b,c^	52.62±0.02^b^	72.70±0.02^b^
>120 ≤ 132	7.92±0.11^a^	1.20±0.02^c,d,e^	9.3±0.21^a^	76.05±0.002^a,b^	56.09±0.001^a,b^	53.14±0.05^a,b^	73.63±0.04^b^
>132 ≤ 144	7.68±0.11^a,b^	1.19±0.02^b,c^	8.33±0.22^a,b^	76.49±0.003^b,c^	56.17±0.001^a,b^	59.37±0.09^a,b^	82.67±0.07^a^
>144 ≤ 156	7.59±0.13^a,b^	0.94±0.03^f^	7.32±0.24^d,e^	76.79±0.003^a,b,c^	56.51±0.001^a^	62.27±0.06^a^	84.61±0.05^a^

VOL=Ejaculate volume in ml; CONC=Sperm concentration (10^9^/ml); TS=Total sperm (10^9^/ejaculate); IM=Initial motility (%); PTM=Post-thaw motility; HOST=Hypo-osmotic swelling test; AIFS=Acrosome integrity of frozen semen.

Means with at least one common superscript within classes do not differ significantly; Significance level -

** p<0.01; *p<0.05; NS=p>0.05.

Sperm concentration of Gir cattle reported by Shelke and Dhami [12] (1.21 × 10^9^) and Chikhaliya *et al*. [15] (1.26 × 10^9^) are in agreement with the results observed in the present study (1.22 × 10^9^). Das *et al*. [10] and Sonar *et al*. [14] observed lower concentrations of 0.70 and 0.94 × 10^9^, respectively, in Gir bulls. The mean of total sperm observed in the present study was lower than total sperm output (9.01 × 10^9^) observed by Pathak *et al*. [11]. However, their study was limited to only three bulls with just 24 ejaculates.

The initial motility percent reported in the present study was in close agreement with the study of Shelke and Dhami [12], while Rana and Dhami [9], Das *et al*. [10], Sannat *et al*. [13], and Sonar *et al*. [14], observed slightly lower motility (69-71%) in Gir cattle. Pathak *et al*. [11] and Chikhaliya *et al*. [15] reported higher (84.71 and 85.83%) motility. The result on post-thaw motility was comparable with the results of Das *et al*. [10] and Pathak *et al*. [11], while Shelke and Dhami [12] reported slightly lower (49.58) values of post-thaw motility. The motilities are measures of subjective evaluation and strongly depend on the evaluator, hence slight variation among different studies is expected.

Observations made on HOST in the present study were in accordance with the findings of Pathak *et al*. [11], while Sonar *et al*. [14] and Chikhaliya *et al*. [15] published higher means (60.12 and 83%) for the trait. Similarly, the mean for acrosome integrity reported by Sonar *et al*. [14] and Chikhaliya *et al*. [15] were higher than the present study. However, the acrosome integrity was measured on fresh semen unlike that of the present study where acrosome integrity was measured on frozen semen. ANOVA for the effect of season of collection indicated significant effect (p<0.01) on the volume, concentration, total sperms, and initial motility of semen of Gir bulls. The means of volume, concentration, and total sperm were found to be higher during the summer season while means for winter and monsoon did not differ from each other. Murphy *et al*. [16] and Fuerst-Waltl *et al*. [17] reported similar results in Holstein Friesian and Austrian Simmental bulls, respectively. The average period of spermatogenesis is 65 days. The influence of seasonal variation during the sensitive stages of spermatogenesis may have effect on semen quality. On the other hand, initial motility was better in winter as compared to summer and monsoon. However, the differences among seasons were too less. Monsoon and summer seasons did not vary much with respect to initial motility. Post-thaw motility, plasma membrane integrity measured by HOST, and acrosome integrity of frozen semen did not show any variation (p>0.05) due to season of collection.

ANOVA for the effect of age at collection showed significant influence (p<0.01) on all semen attributes in the present study. The gradual increment in the volume was observed up to 120 months and later declined in older bulls. Similar trend of volume was followed by total sperm count. The trend of initial motility was inconsistent but showed a slight decreasing tendency with age. Fuerst-Waltl *et al*. [17] and Mathevon *et al*. [18] found similar findings for volume and total sperm in exotic breeds (Holstein and Austrian Simmental). Decrease in initial motility observed in old bulls was also reported by Brito *et al*. [2]. The ejaculate volume and sperm concentration showed opposite trends as the age progressed. Trend observed in post-thaw motility was not consistent with age. There was gradual improvement in HOST reactive sperm and acrosomal integrity over age till 96 months after which the performance with respect to these traits declined with lowest values at 120 months of age. However, increase in these qualitative traits observed in the older age classes from 132 to 156 months was unexpected in plasma membrane and acrosome integrity. The possible reason for this increase could be that by the end of 120 months, 84% of bulls were culled from the herd, and the improved performance of the bulls retained after preferential culling where the bull with high demand in the field (possibly due to better fertility) was kept for an extended period. Longitudinal data analysis was carried out for all repeatable traits. It was found that the random effect of age within bull was highly significant (with “p” value almost zero). It indicated the presence of between bull variation in traits within age and each age group had different intercepts. The acrosome integrity of frozen semen and HOST showed positive trend, which showed progressive increase in fertility with increase in age at collection, while other traits indicated progressive decrease with increase in age at collection.

The genetic parameters for all semen attributes are presented in Table-2. The repeatability of semen traits ranged from 0.04 (low) to 0.58 (high). The lowest values were observed for post-thaw motility and HOST. Moderate repeatability estimates were observed in semen concentration, total sperm, and initial motility parameters, while higher repeatability estimates were observed for volume and acrosome integrity. The bull effect correlation is combination of genetic and permanent environmental variation. The bull effect correlation ranged from −0.74 to 0.93. A bull effect of almost unity was observed between HOST and acrosome integrity. Apart from that, higher positive bull effects were observed for volume and concentration with total sperm followed by volume and total sperm with initial motility. Higher negative bull effects were observed for HOST and acrosome integrity with initial motility followed by HOST and acrosome integrity with total sperm. A low positive bull effect was observed between volume and concentration; however, the standard errors were high due to lesser number of bulls involved in the study.

**Table-2:** Bull effects (Genetic + Permanent environmental correlation; above diagonal values), phenotypic correlation (below diagonal values), and repeatability estimates (diagonal values) of semen traits of Gir bulls.

Trait	VOL	CONC	TS	IM	PTM	HOST	AIFS
VOL	0.46±0.06	0.14±0.19	0.79±0.07	0.63±0.11	0.26±0.18	−0.41±0.32	−0.50±0.15
CONC	0.09±0.06	0.20±0.04	0.71±0.10	0.26±0.18	0.48±0.16	−0.24±0.40	−0.23±0.21
TS	0.54±0.05	0.61±0.03	0.32±0.05	0.66±0.11	0.44±0.17	−0.48±0.36	−0.54±0.15
IM	0.26±0.06	0.11±0.04	0.25±0.05	0.29±0.05	0.21±0.19	−0.68±0.35	−0.73±0.10
PTM	0.05±0.04	0.05±0.03	0.07±0.03	0.06±0.03	0.09±0.03	0.22±0.39	0.01±0.22
HOST	−0.01±0.05	−0.09±0.06	−0.15±0.05	−0.11±0.06	0.05±0.05	0.04±0.03	0.93±0.29
AIFS	−0.28±0.09	−0.15±0.08	−0.19±0.08	−0.31±0.07	0.06±0.06	0.20±0.05	0.58±0.07

VOL=Ejaculate volume in ml; CONC=Sperm concentration (10^9^/ ml); TS=Total sperm (10^9^/ejaculate); IM=Initial motility (%); PTM=Post-thaw motility (%); HOST=Hypo-osmotic swelling test (%); AIFS=Acrosome integrity of frozen semen (%)

Phenotypic correlation ranged from −0.31 (initial motility and acrosome integrity) to 0.61 (concentration and total sperm). The estimates of repeatability and bull effect correlation could be regarded as a higher boundary of heritability and genetic correlation of the respective traits. The estimates reported in the present study show that the future performance of a bull can be explained based on initial values with some accuracy for ejaculate volume and acrosome integrity of frozen semen. A thorough search of literature indicated lack of studies on genetic parameters of semen traits in Gir bulls. However, few researchers presented phenotypic correlation among the semen traits in Gir bulls [11, 15] and the values observed were comparable with the present study. Pathak *et al*. [11] reported low negative (−0.07) correlation while Chikhaliya *et al*. [15] presented low positive correlation (0.026) between volume and concentration. The same researchers found low (0.11 and 0.23) phenotypic correlation between volume and initial motility and moderate (0.030 and 0.34) correlation between concentration and initial motility.

## Conclusion

It is concluded that seasonal variations in semen parameters were due to the influence of climatic condition during the sensitive stages of spermatogenesis. The age-related trend in semen attributes observed in the present study indicated that individual bulls showed variation in traits measured over age at collection. The estimates of repeatability and bull effects may be helpful in explaining the future performances of bulls with some accuracy. In the end, knowledge of the genetic and environmental factors influencing semen production can help an AI stud to achieve a high semen production of good quality with desired profit.

## Authors’ Contributions

KGB analyzed the data, prepared, and edited the manuscript. KTPJ and RV helped in designing the methodology and models, and conceptualizing the research work. RV, PK, TS, and CJ reviewed and edited the manuscript. PD processed the semen samples and helped in data collection. TSh helped in data retrieval and computer programing. All the authors read and approved the final copy of the manuscript.

## Competing Interests

The authors declare that they have no competing interests.

## Publisher’s Note

Veterinary World remains neutral with regard to jurisdictional claims in published institutional affiliation.

## References

[1] Department of Animal Husbandry and Dairying, Government of India (2019). Animal Husbandry Statistics Division. Livestock Census.

[2] Brito L.F.C, Silva A.E.D, Rodrigues L.H, Vieira F.V, Deragon L.A.G, Kastelic J.P. (2002). Effects of environmental factors, age and genotype on sperm production and semen quality in *Bos indicus* and *Bos taurus* AI bulls in Brazil. Anim. Reprod. Sci.

[3] Bhakat M, Mohanty T.K, Raina V.S, Gupta A.K, Khan H.M, Mahapatra R.K, Sarkar M. (2011). Effect of age and season on semen quality parameters in Sahiwal bulls. Trop. Anim. Health Pro.

[4] Tiwari R, Mishra G.K, Singh R.B, Rehman S.U, Rathora K.S, Saxena S.K, Siddiqui M.U. (2012). Seasonal variations in the quality and freezability of Red Sindhi bull semen. Indian J. Anim. Sci.

[5] Iliger M.R (2014). Studies on Semen Production Potential of Amritmahal Bulls M. V. SC. Dissertation.

[6] Government of India (2012). Minimum Standards for Production (MSP) of Bovine Frozen Semen. Department of Animal Husbandry, Dairying and Fisheries, Ministry of Agriculture, Government of India, India. http://www.dahd.nic.in..

[7] R Core Team (2019). R: A Language and Environment for Statistical Computing.

[8] Meyer K (2007). WOMBAT-a tool for mixed model analyses in quantitative genetics by restricted maximum likelihood (REML). J. Zhejiang Univ. Sci. B.

[9] Rana C.M, Dhami A.J. (2004). Physical attributes, intact acrosome, HOS test and freezability of semen of Gir and Jafarabadi bulls. Indian Vet. J.

[10] Das S, Sana D, Gupta T, Chowdhury S, Chakraborti A, Biswas J, Sarkar S. (2017). Seasonal variation in semen production parameters of Gir bulls reared under tropical climate. IJAR.

[11] Pathak P.K, Dhami A.J, Chaudhari D.V. (2018). Seminal attributes, freezability and their interrelationships in zebu cattle and buffalo bulls from central Gujarat. IJVSBT.

[12] Shelke V.B, Dhami A.J. (2001). Comparative evaluation of physico-morphological attributes and freezability of semen of Gir cattle (*Bos indicus*) and Jafarabadi buffalo (*Bubalus bubalis*) bulls. Indian J. Anim. Sci.

[13] Sannat C, Nair A, Sahu S.B, Sahasrabudhe S.A, Kumar A, Gupta A.K, Shende R.K. (2015). Effect of species, breed, and age on bacterial load in bovine and bubaline semen. Vet. World.

[14] Sonar B.P, Tiwari R.P, Poyam M.R, Mishra G.K, Pandey A.K, Nair A.K, Sahasrabudhe S.A. (2016). Characteristics and freezability of Gir bull semen. Indian J. Anim. Sci.

[15] Chikhaliya P.S, Ahlawat A.R, Solanki G.S, Raval R.J, Vala K.B, Verma A.D. (2018). Physical seminal attributes of Gir bull semen. Int. J. Curr. Microbiol. App. Sci.

[16] Murphy E.M, Kelly A.K, O'Meara C, Eivers B, Lonergan P, Fair S. (2018). Influence of bull age, ejaculate number, and season of collection on semen production and sperm motility parameters in Holstein Friesian bulls in a commercial artificial insemination centre. J. Anim. Sci.

[17] Fuerst-Waltl B, Schwarzenbacher H, Perner C, Sölkner J. (2006). Effects of age and environmental factors on semen production and semen quality of Austrian Simmental bulls. Anim. Reprod. Sci.

[18] Mathevon M, Buhr M.M, Dekkers J.C.M. (1998). Environmental, management, and genetic factors affecting semen production in Holstein bulls. J. Dairy Sci.

